# Supplementary material for: Decoding polyubiquitin regulation of KV7. 1 (KCNQ1) surface expression with engineered linkage-selective deubiquitinases
Source: Nat Commun. 2025 Jul 1;16:5805. doi: 10.1038/s41467-025-60893-0 (PMC12218124; doi:10.1038/s41467-025-60893-0)
Supplement: Supplementary file 1 — Supplementary Information [file 41467_2025_60893_MOESM1_ESM.pdf]

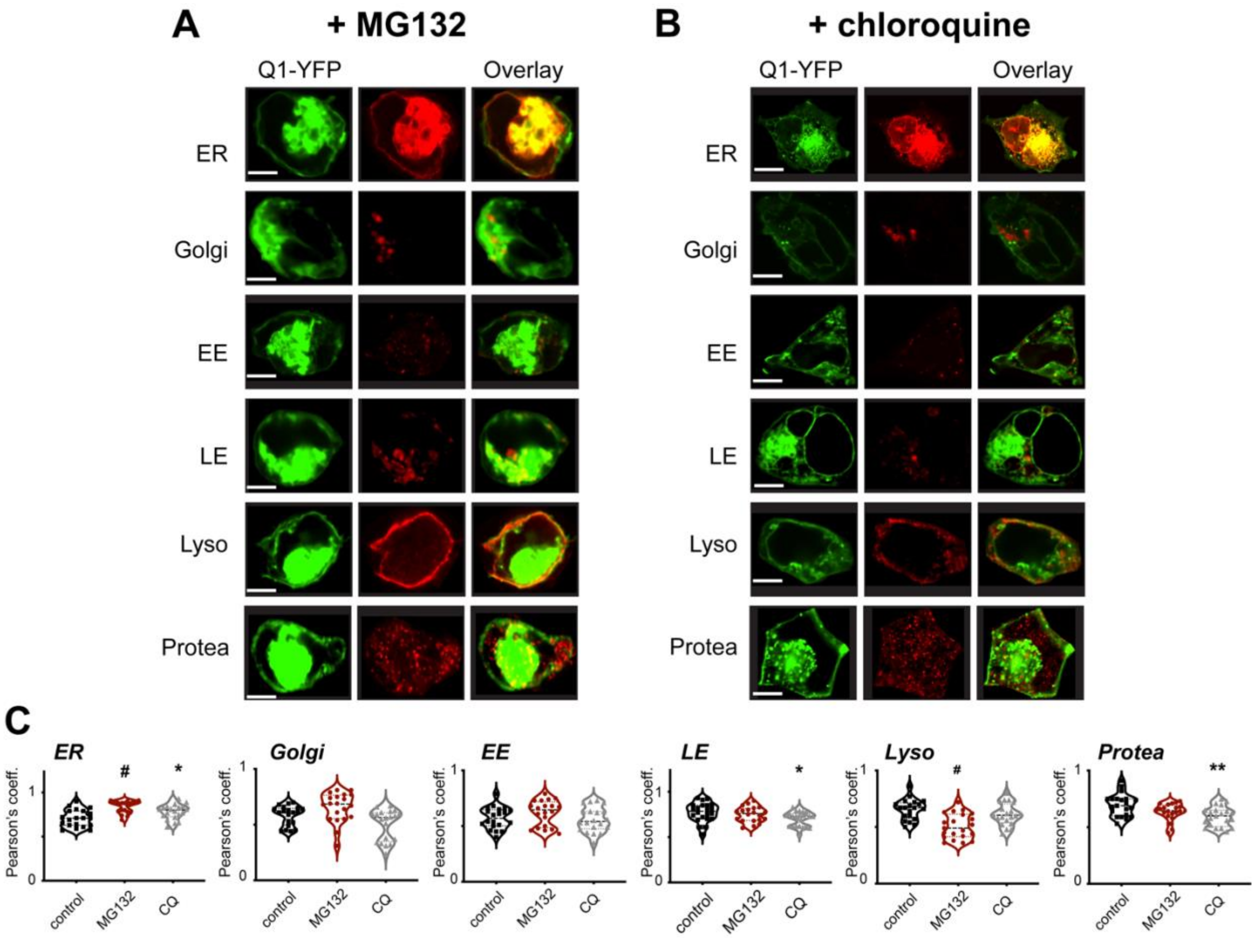

Supplemental Figure 1: KCNQ1-YFP subcellular distribution under proteasomal and lysosomal inhibition.

(A) Representative confocal images of HEK293 cells expressing KCNQ1-YFP (green), treated with MG132, and with immunolabelled subcellular organelles (red) [calnexin - endoplasmic reticulum (ER); RCAS1 – Golgi; EEA1 - early endosome (EE); Rab9A - late endosome (LE); LAMP2 - lysosome (lyso); and PSMA2 - proteasome (protea)]. Scale bar, 5 μm (B) Representative confocal images of HEK293 cells expressing KCNQ1-YFP (green), treated with chloroquine, and with immunolabelled subcellular organelles (red) [calnexin - endoplasmic reticulum (ER); RCAS1 – Golgi; EEA1 - early endosome (EE); Rab9A - late endosome (LE); LAMP2 - lysosome (lyso); and PSMA2 - proteasome (protea)]. (C) Pearson's correlational coefficient of KCNQ1-YFP colocalization with the subcellular organelles ER, Golgi, EE, LE, lysosomes and proteasomes (*n*>20; one-way ANOVA with Dunnett's multiple comparisons tests, **p*<0.05, ***p*<0.01 and $^{\#}p$<0.0001).

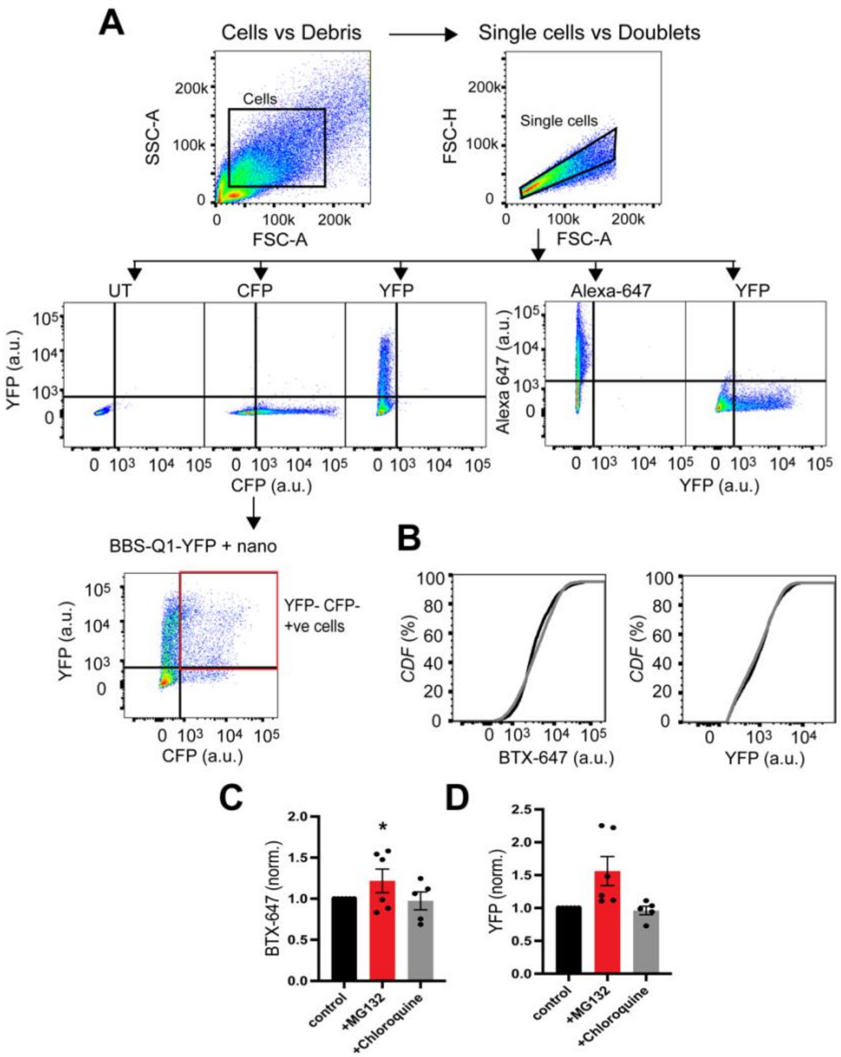

Supplemental Figure 2: Impact of MG132 and chloroquine on KCNQ1-YFP abundance and surface density

(A) Gating strategy used to determine cells vs debris (FSC/SSC) and single cells vs doublets (FSC-A/FSC-H) here and throughout. Fluorescence gates (CFP and YFP) were determined from single color controls compared to untransfected cells. Cells in CFP- YFP- positive quadrant were selected for analysis here and throughout. (B) Representative flow cytometry CDF plot showing surface (BTX_{647}) fluorescence (left) and total (YFP) fluorescence in cells expressing KCNQ1-YFP under untreated (black) and chloroquine treated (gray) conditions. (C) Quantification of flow cytometry experiments for KCNQ1-YFP surface expression (BTX-647 fluorescence) analyzed from YFP- positive cells in untreated (black), MG132 (red) and chloroquine (gray) treated conditions (*n*>5,000 cells per experiments; *N*=5-6; *$p<0.05$, one-way ANOVA). Data were normalized to the control group. (D) Quantification of flow cytometry experiments for KCNQ1-YFP total expression (YFP fluorescence) analyzed from YFP- positive cells in untreated (black), MG132 (red) and chloroquine (gray) treated conditions (*n*>5,000 cells per experiments; *N*=5-6; *ns*, $p>0.05$, one-way ANOVA). Data were normalized to the control group.

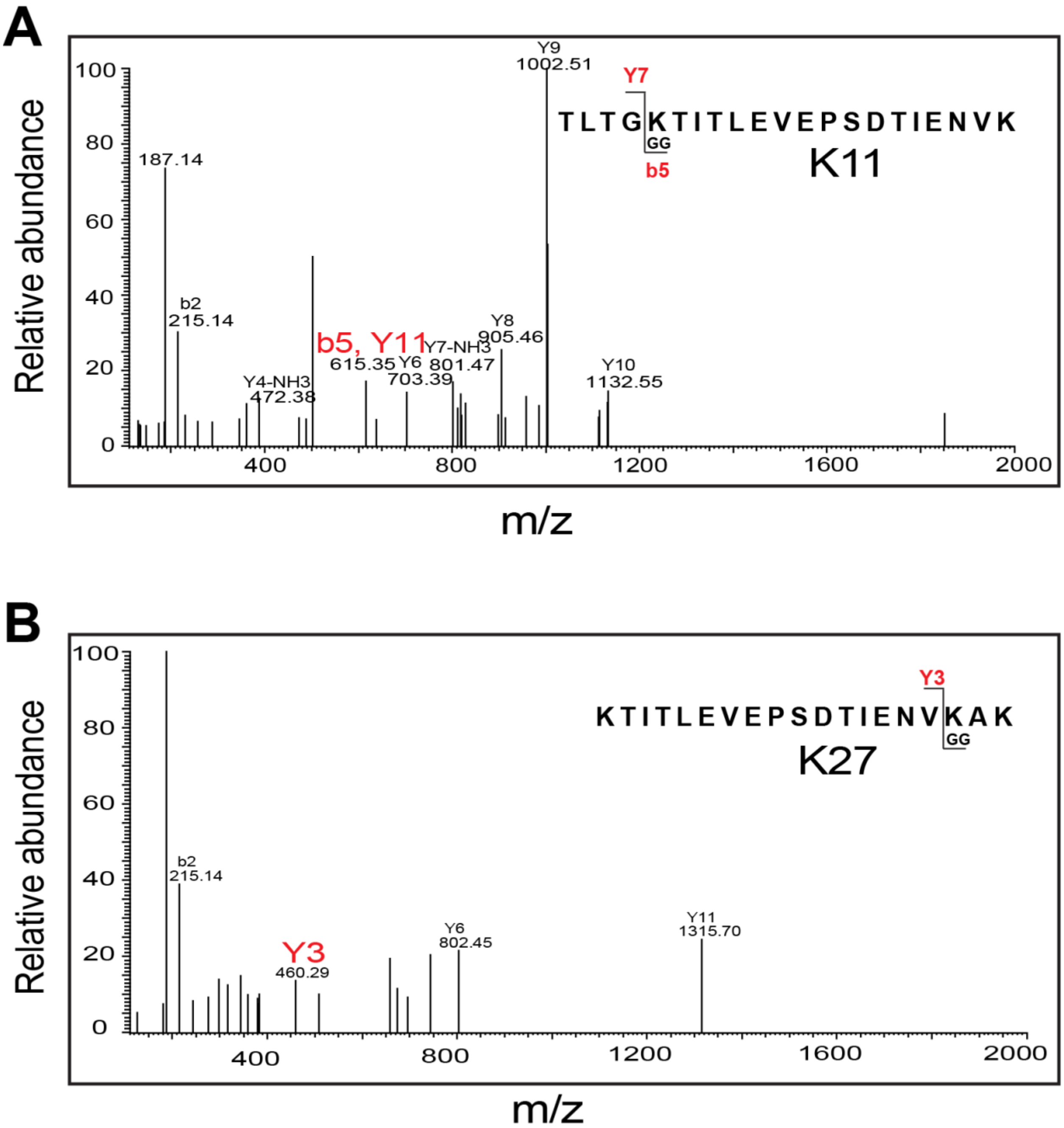

Supplemental Figure 3: Exemplar ms2 spectra

(A) Exemplar ms2 spectra traces for K11 and (B) K27 ubiquitin digly peptides.

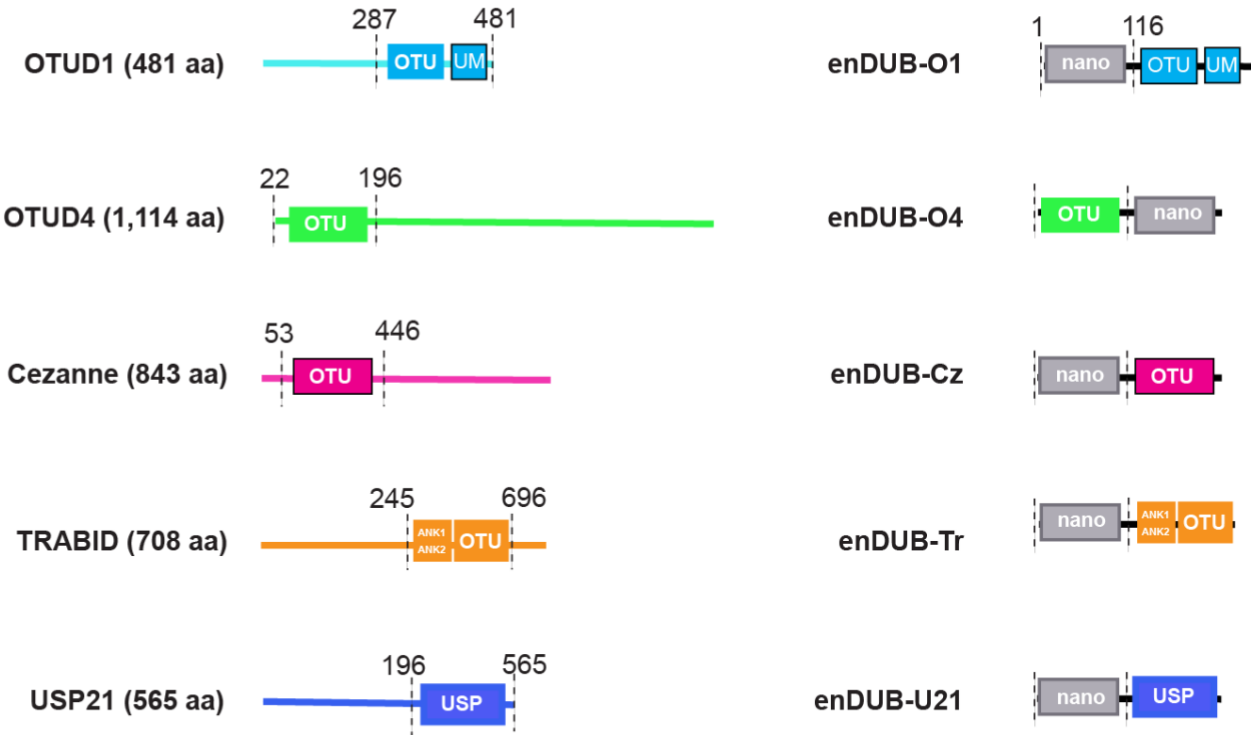

Supplemental Figure 4: Design of linkage-selective and non-specific enDUBs

Schematic of enDUB design.

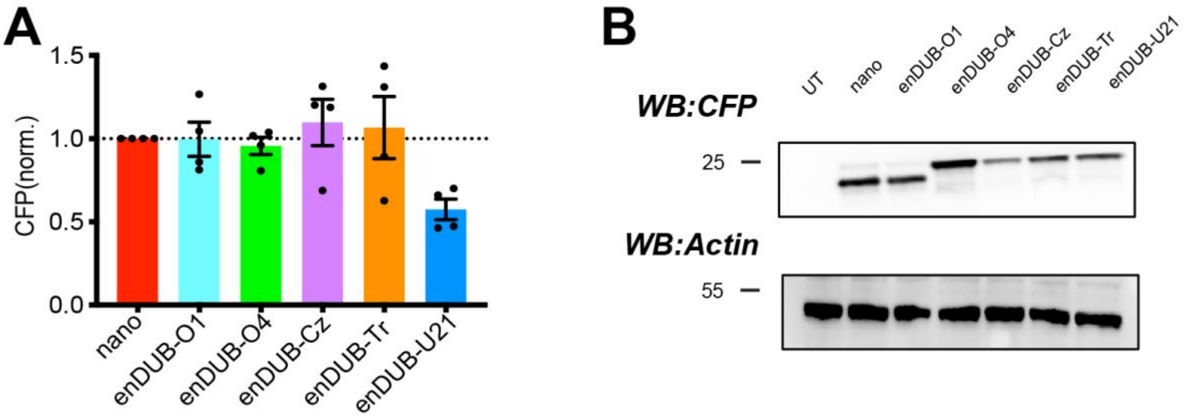

Supplemental Figure 5: enDUB expression.

(A) Quantification of flow cytometry experiments for nano/enDUB expression analyzed from CFP- and YFP- positive cells (n>5,000 cells per experiments; *N*=4; One-way ANOVA with Dunnett's multiple comparisons). Data were normalized to the values from the nano control group (dotted line). (B) *Top*, Western blot analysis of CFP expression in cells expressing KCNQ1-YFP with either nano or one of the enDUBs. Different sizes of the CFP band is due to whether nano/DUB is upstream or downstream of the P2A-CFP expression cassette; *bottom*, same blot probed for actin.

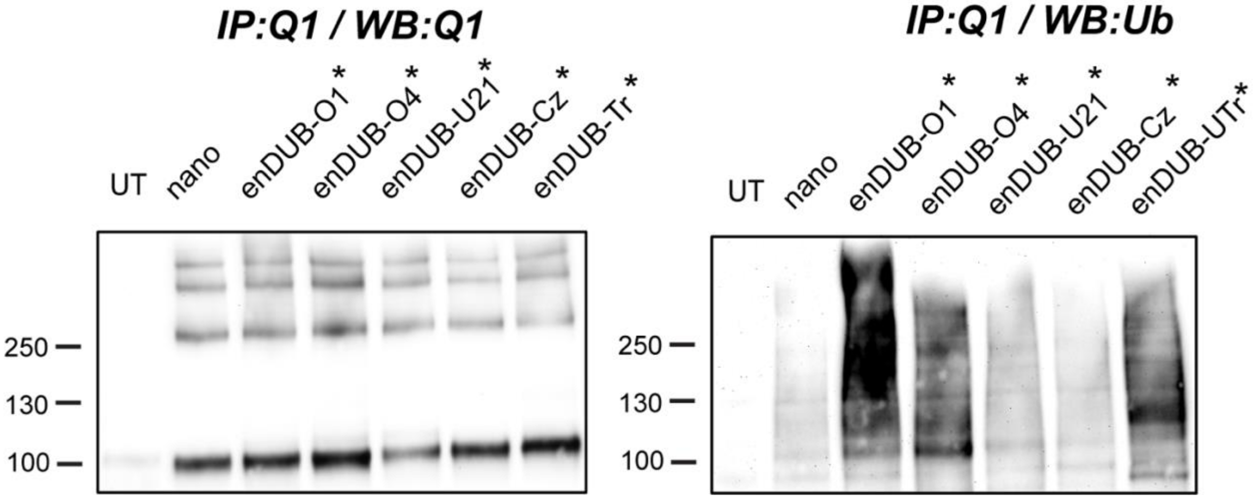

Supplemental Figure 6: Catalytic activity of enDUBs is essential for their impact on KCNQ1-YFP ubiquitination.

Representative blot of immunoprecipitated KCNQ1-YFP co-expressed with the catalytically dead (*) enDUBs and probed with anti-KCNQ1 (left). The same blot was stripped and probed again with anti-ubiquitin (right).

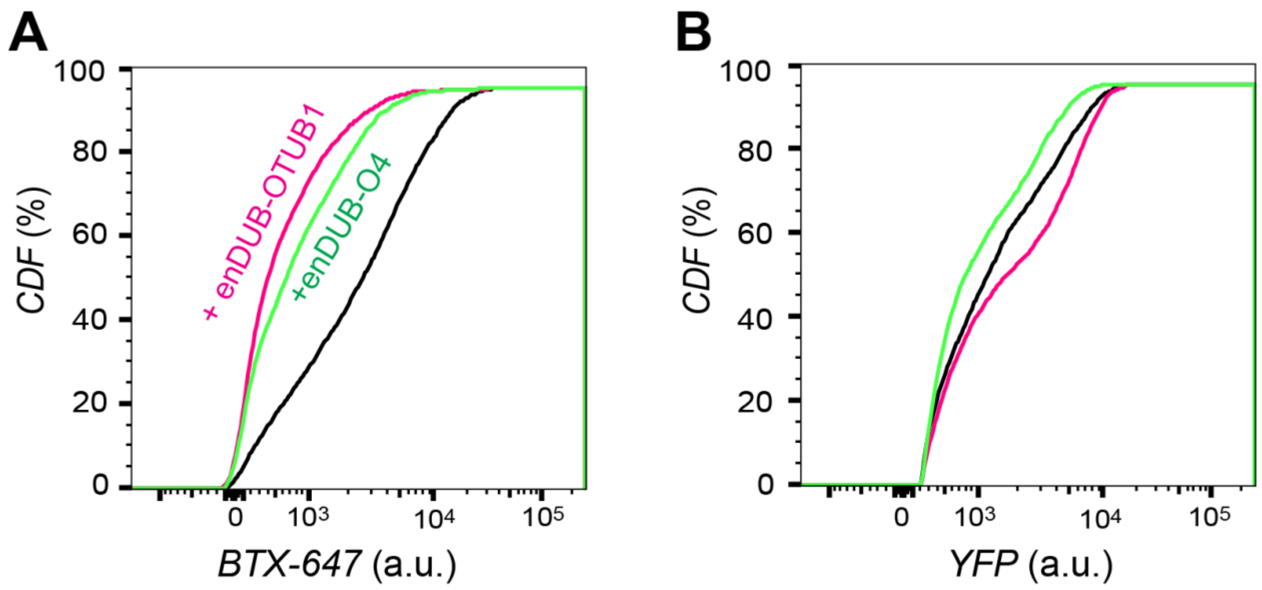

Supplemental Figure 7: Downregulation of KCNQ1-YFP surface density by distinct K48-selective enDUBS.

(A) Representative flow cytometry CDF plot showing surface (BTX-647) fluorescence (B) and total (YFP) fluorescence in cells expressing KCNQ1-YFP with either nano (black), enDUB-O4 (green), or enDUB-OTUB1 (pink).

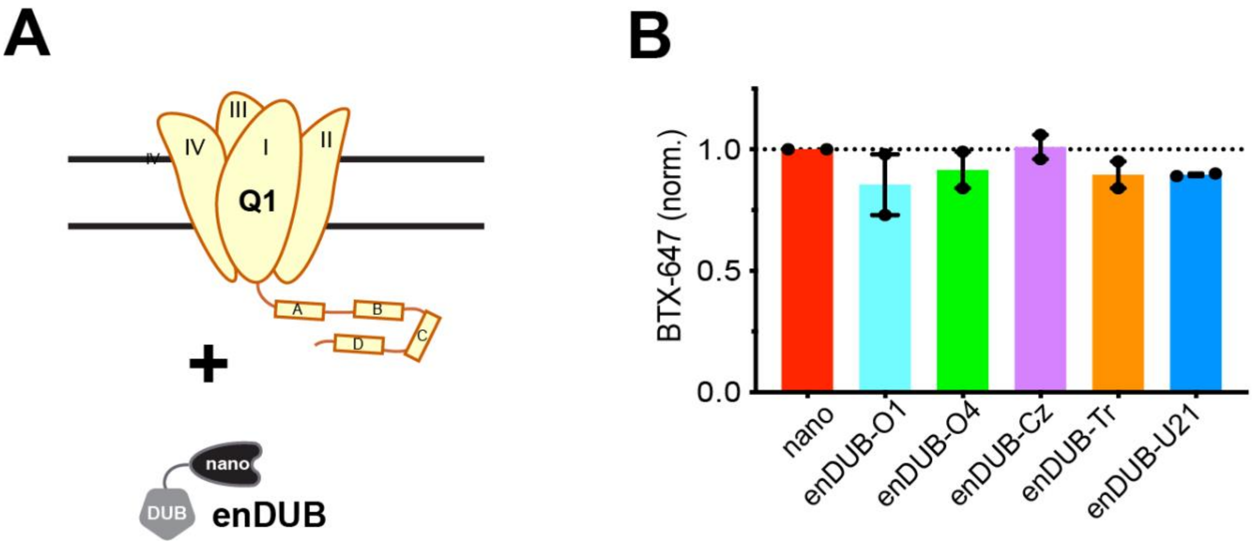

Supplemental Figure 8: GFP/YFP-targeted enDUBs have minimal impact on surface density of BBS-KCNQ1 that lacks a YFP tag.

(A) Scheme. BBS-KCNQ1 co-expressed with the enDUBs. (B) Quantification of flow cytometry experiments for BBS-KCNQ1 surface expression analyzed from YFP- CFP- positive cells (n > 5,000 cells per experiments; *N*=2). Data were normalized to the values from the nano control group (dotted line).

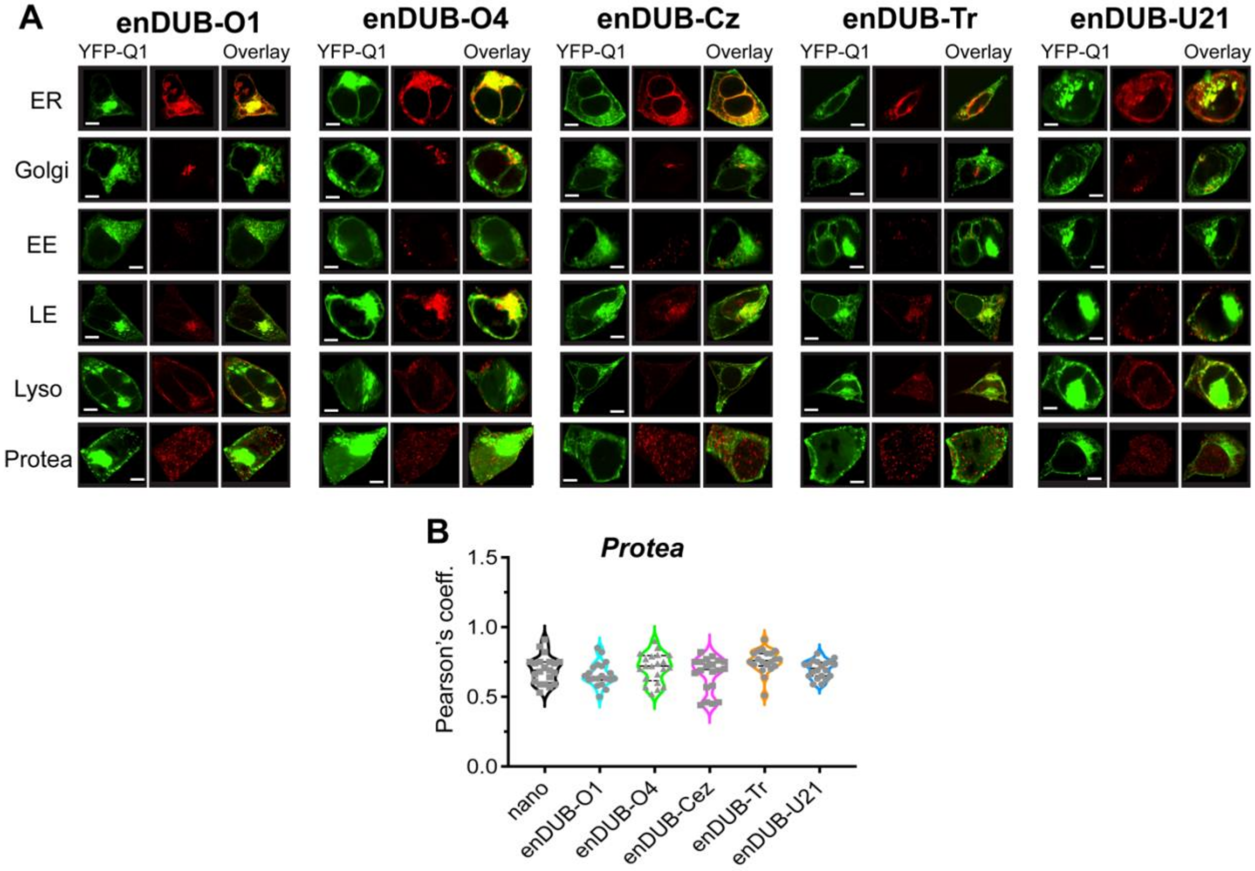

Supplemental Figure 9: Confocal images and quantification of subcellular redistribution of KCNQ1-YFP by the enDUBs.

(A) Exemplar confocal images of HEK293 cells expressing KCNQ1-YFP (green) and enDUBs with immunostaining of the subcellular organelles (red) [calnexin - endoplasmic reticulum (ER); RCAS1 – Golgi; EEA1 - early endosome (EE); Rab9A - late endosome (LE); LAMP2 - lysosome (lyso); and PSMA2 - proteasome (protea)]. Scale bar, 5 μm. (B) Co-localization of KCNQ1-YFP with proteasomal subcellular marker (PSMA2) assessed by Pearson's co-localization coefficient (*N*=3, *n*>20; n.s., one-way ANOVA and Dunnett's multiple comparisons test).

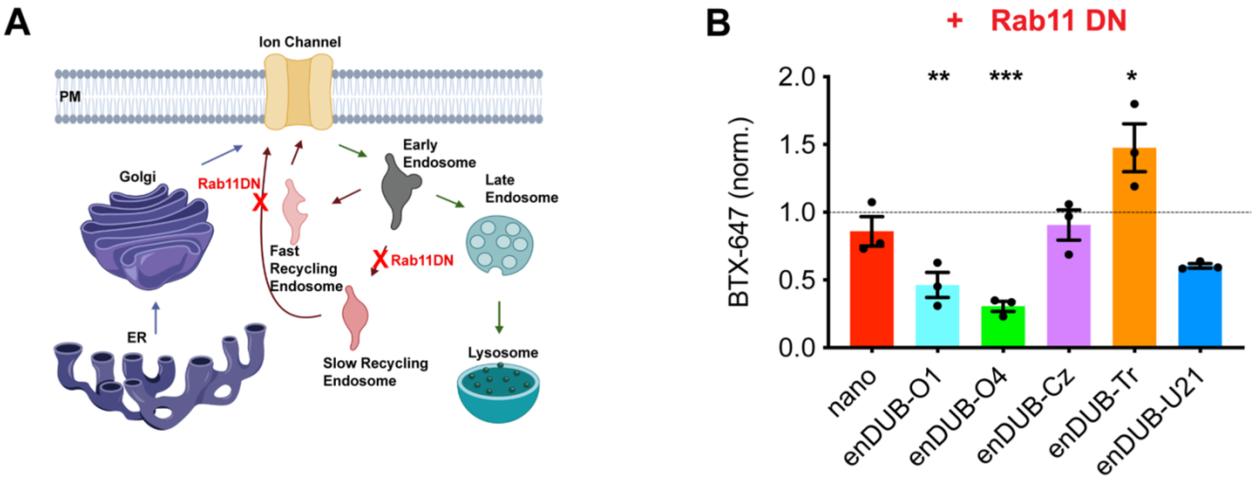

Supplemental Figure 10: Differential impact of the enDUBs on KCNQ1-YFP surface density in the presence of Rab11DN.

(A) Cartoon of impact of Rab11DN mediated recycling of KCNQ1-YFP. Created in BioRender. Shanmugam, S. (2025) https://BioRender.com/6718cdu. (B) Quantification of flow cytometry experiments for KCNQ1 surface expression analyzed from YFP- positive cells (n>5,000 cells per experiments; N=3; ***p<0.001, **p<0.001 and *p<0.05, one-way ANOVA with Dunnett's multiple comparisons). Data were normalized to the values from the nano control group (dotted line).

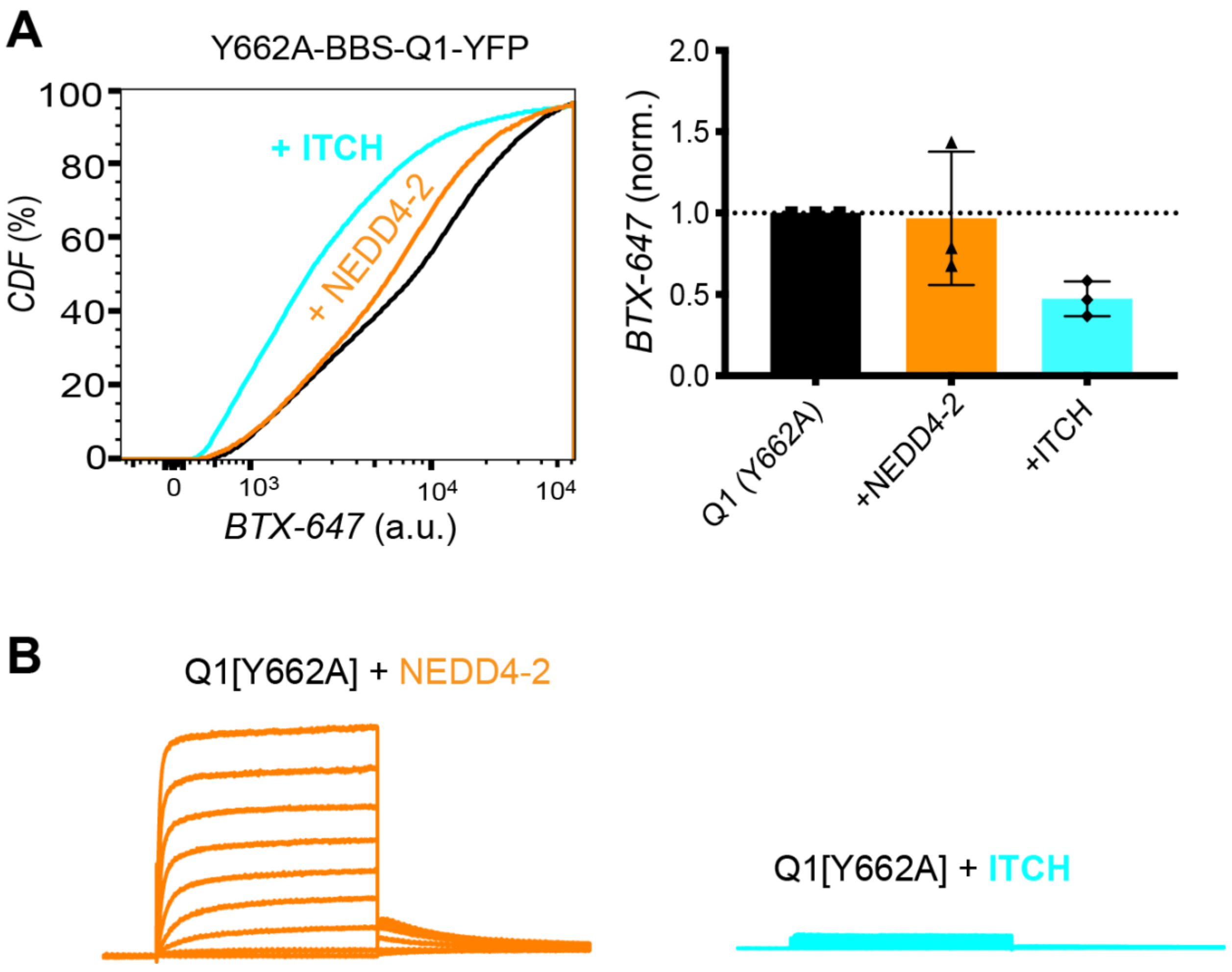

Supplemental Figure 11: Differential effect of NEDD4-2 and ITCH on KCNQ1 PY mutant.

(A) Representative flow cytometry CDF plot showing surface (BTX-647) fluorescence in cells expressing Y662-KCNQ1-YFP NEDD4-2 and ITCH. Quantification of flow cytometry experiments for KCNQ1-YFP surface expression analyzed from YFP- positive cells (n>5,000 cells per experiments; *N*=3). Data were normalized to the values from the nano control group (dotted line). (B) Exemplar Y662A KCNQ1-YFP current traces from whole-cell patch clamp measurements in CHO cells with NEDD4-2 (orange) and ITCH (cyan).

MAAASSPPRAERKRWGWGRLPGARRGSAGLAKKCPFSLELAEG
GPAGGALYAPIAPGAPGPAPPASPAAPAAPPVASDLGPRPPVS
LDPRVSIYSTRRPVLARTHVQGRVYNFLERPTGWKCFVYHFAV
FLIVLVCLIFSVLSTIEQYAALATGTLFWMEIVLVVFFGTEYV
VRLWSAGCRSKYVGLWGRLRFARKPISIIDLIVVVASMVVLCV
GSKGQVFATSAIRGIRFLQILRMLHVDRQGGTWRLLGSVVFIH
RQELITTLYIGFLGLIFSSYFVYLAEKDAVNESGRVEFGSYAD
ALWWGVVTVTTIGYGDKVPQTWVGKTIASCFSVFAISFFALPA
GILGSGFALKVQQKQRQKHFNRQIPAAASLIQTAWRCYAAENP
DSSTWKIYIRKAPRSHTLLSPSPKPKKSVVVKKKKFKLDKDNG
VTPGEKMLTVPHITCDPPEERRLDHFSVDGYDSSVRKSPTLLE
VSMPHFMRTNSFAEDLDLEGETLLTPITHISQLREHHRATIKV
IRRMQYFVAKKKFQQARKPYDVRDVIEQYSQGHLNLMVRIKEL
QRRLDQSIGKPSLFISVSEKSKDRGSNTIGARLNRVEDKVTQL
DQRLALITDMLHQLLSLHGGSTPGSGGPPREGGAHITQPCGSG
GSVDPELFLPSNTLPTYEQLTVPRRGPDEGS

PY motif

Supplemental Figure 12: KCNQ1 sequence.

Sequence of KCNQ1 channel. Cytosolic regions are highlighted in green, transmembrane segments are in blue, extracellular regions are in gray, PY motif is in maroon. Cytosolically accessible unmodified and modified (as detected by mass spectrometry) lysine (K) residues are highlighted in pink and in yellow, respectively.
