# Supplementary material for: Decoding polyubiquitin regulation of KV7. 1 (KCNQ1) surface expression with engineered linkage-selective deubiquitinases
Source: Nat Commun. 2025 Jul 1;16:5805. doi: 10.1038/s41467-025-60893-0 (PMC12218124; doi:10.1038/s41467-025-60893-0)
Supplement: Supplementary file 2 — Reporting Summary [file 41467_2025_60893_MOESM2_ESM.pdf]

nature portfolio

Corresponding author(s): Henry M. Colecraft

Last updated by author(s): May 23, 2025

Reporting Summary

Nature Portfolio wishes to improve the reproducibility of the work that we publish. This form provides structure for consistency and transparency in reporting. For further information on Nature Portfolio policies, see our Editorial Policies and the Editorial Policy Checklist.

Statistics

For all statistical analyses, confirm that the following items are present in the figure legend, table legend, main text, or Methods section.

n/a	Confirmed	
☐	☒	The exact sample size (*n*) for each experimental group/condition, given as a discrete number and unit of measurement
☐	☒	A statement on whether measurements were taken from distinct samples or whether the same sample was measured repeatedly
☐	☒	The statistical test(s) used AND whether they are one- or two-sided<br>*Only common tests should be described solely by name; describe more complex techniques in the Methods section.*
☐	☒	A description of all covariates tested
☐	☒	A description of any assumptions or corrections, such as tests of normality and adjustment for multiple comparisons
☐	☒	A full description of the statistical parameters including central tendency (e.g. means) or other basic estimates (e.g. regression coefficient) AND variation (e.g. standard deviation) or associated estimates of uncertainty (e.g. confidence intervals)
☐	☒	For null hypothesis testing, the test statistic (e.g. *F*, *t*, *r*) with confidence intervals, effect sizes, degrees of freedom and *P* value noted<br>*Give P values as exact values whenever suitable.*
☒	☐	For Bayesian analysis, information on the choice of priors and Markov chain Monte Carlo settings
☒	☐	For hierarchical and complex designs, identification of the appropriate level for tests and full reporting of outcomes
☐	☒	Estimates of effect sizes (e.g. Cohen's *d*, Pearson's *r*), indicating how they were calculated

Our web collection on statistics for biologists contains articles on many of the points above.

Software and code

Policy information about availability of computer code

Data collection	BD FACSDiva (v8.01); NIS-Elements (v5.02); HEKA Pulse (v8.65)
Data analysis	FlowJo (vl0.6.0); FIJI/ImageJ (2.0.0-rc-69/1.Slo); GraphPad Prism 7; Microsoft Excel (v16.27); Origin (v9.7); HEKA FITMaster (v2x73.5).

For manuscripts utilizing custom algorithms or software that are central to the research but not yet described in published literature, software must be made available to editors and reviewers. We strongly encourage code deposition in a community repository (e.g. GitHub). See the Nature Portfolio guidelines for submitting code & software for further information.

Data

Policy information about availability of data

All manuscripts must include a data availability statement. This statement should provide the following information, where applicable:

- Accession codes, unique identifiers, or web links for publicly available datasets
- A description of any restrictions on data availability
- For clinical datasets or third party data, please ensure that the statement adheres to our policy

The authors declare that all data supporting the findings of this study are available within the paper and its supplementary information files. Source data are provided with this paper. The mass spectrometry proteomics data have been deposited to the ProteomeXchange Consortium via the PRIDE partner repository with the dataset identifier PXD057805. Project Name: Digly analysis of human Kv7.1 transfected in HEK293 cells

Research involving human participants, their data, or biological material

Policy information about studies with human participants or human data. See also policy information about sex, gender (identity/presentation), and sexual orientation and race, ethnicity and racism.

Reporting on sex and gender	n/a
Reporting on race, ethnicity, or other socially relevant groupings	n/a
Population characteristics	n/a
Recruitment	n/a
Ethics oversight	n/a

Note that full information on the approval of the study protocol must also be provided in the manuscript.

Field-specific reporting

Please select the one below that is the best fit for your research. If you are not sure, read the appropriate sections before making your selection.

- [x] Life sciences
- [ ] Behavioural & social sciences
- [ ] Ecological, evolutionary & environmental sciences

For a reference copy of the document with all sections, see nature.com/documents/nr-reporting-summary-flat.pdf

Life sciences study design

All studies must disclose on these points even when the disclosure is negative.

Sample size	The sample size of experiments was based on previous experience and published studies (e.g. Morgenstern et al, 2019, elife). The sample sizes are robust enough based on the magnitude of the observed differences and consistency of the various measurements.
Data exclusions	For whole-cell electrophysiological experiments cells were excluded if the seal resistance <200 Mohm resulting in a large basal leak current (heterologous cells and ventricular cardiomyocytes). These are pre-established criteria from years of experience with electrophysiological experiments.
Replication	Experiments had at least 3 independent successful replicates.
Randomization	No randomization was applied based on the types of experiments. Test and control groups were assessed in parallel and isochronally.
Blinding	No blinding was applied due to the nature of the experiments. The most effective process was for one experimentalist to prepare the cells and conduct the experiments. As such, they were aware of the different control and test conditions.

Reporting for specific materials, systems and methods

We require information from authors about some types of materials, experimental systems and methods used in many studies. Here, indicate whether each material, system or method listed is relevant to your study. If you are not sure if a list item applies to your research, read the appropriate section before selecting a response.

Materials & experimental systems

n/a	Involved in the study	
[ ]	[x]	Antibodies
[ ]	[x]	Eukaryotic cell lines
[x]	[ ]	Palaeontology and archaeology
[ ]	[x]	Animals and other organisms
[x]	[ ]	Clinical data
[x]	[ ]	Dual use research of concern
[x]	[ ]	Plants

Methods

n/a	Involved in the study	
[x]	[ ]	ChIP-seq
[ ]	[x]	Flow cytometry
[x]	[ ]	MRI-based neuroimaging

Antibodies

Antibodies used	Calnexin (Abcam - Ab22595), RCAS1 (Cell signaling Tech - D2B6N), Rab9A (Cell signaling Tech - D52G8), EEA1 (Cell signaling Tech - C45B10), LAMP2 (Sigma Aldrich - L0668), KCNQ1 (Alomone labs - APC-022) and ubiquitin (LifeSensors - VU1).

Validation	All antibodies were validated by binding assays by manufacturer.

Eukaryotic cell lines

Policy information about cell lines and Sex and Gender in Research

Cell line source(s)	Human embryonic kidney (HEK293) cells were obtained from ATCC.
Authentication	HEK293 cells were authenticated via STR analysis (ATCC) with exact match for ATCC CRL-1573.
Mycoplasma contamination	Cells were mycoplasma free, as determined by the MycoFluor Mycoplasma Detection Kit (Invitrogen).
Commonly misidentified lines (See ICLAC register)	No commonly misidentified cell lines were used.

Animals and other research organisms

Policy information about studies involving animals; ARRIVE guidelines recommended for reporting animal research, and Sex and Gender in Research

Laboratory animals	Adult male Hartley guinea pigs (Charles River) , 250 g, 4 weeks old.
Wild animals	n/a
Reporting on sex	We isolated cardiomyocytes from adult male Hartley guinea pigs.
Field-collected samples	*For laboratory work with field-collected samples, describe all relevant parameters such as housing, maintenance, temperature, photoperiod and end-of-experiment protocol OR state that the study did not involve samples collected from the field.*
Ethics oversight	Experiments were performed in accordance with the guidelines of Columbia University Animal Care and Use Committee (IACUC).

Note that full information on the approval of the study protocol must also be provided in the manuscript.

Plants

Seed stocks	n/a
Novel plant genotypes	n/a
Authentication	n/a

Flow Cytometry

Plots

Confirm that:

- [x] The axis labels state the marker and fluorochrome used (e.g. CD4-FITC).
- [x] The axis scales are clearly visible. Include numbers along axes only for bottom left plot of group (a 'group' is an analysis of identical markers).
- [x] All plots are contour plots with outliers or pseudocolor plots.
- [x] A numerical value for number of cells or percentage (with statistics) is provided.

Methodology

Sample preparation	Samples were prepared as described in our methods section (flow cytometry).
Instrument	BD LSRII Cell Analyzer (BD Biosciences, San Jose, CA, USA)
Software	BD FACSDiva was used to collect data. FlowJo (vl0.10.0) was used to analyze surface labeling assays.
Cell population abundance	70% of cells were analyzed, gating for live single cells.

Gating strategy	Appropriate gates were used to determine cells vs debris (FSC/SSC) and single cells vs doublets (FSC-A/FSC-H). Subsequent gates were determined with single color controls, and values were analyzed from co-transfected cell populations (YFP- and CFP-positive as described in our manuscript).

☒ Tick this box to confirm that a figure exemplifying the gating strategy is provided in the Supplementary Information.
